# Transcriptome Analysis Identifies Candidate Genes and Pathways Associated With Feed Efficiency in Hu Sheep

**DOI:** 10.3389/fgene.2019.01183

**Published:** 2019-11-19

**Authors:** Deyin Zhang, Xiaoxue Zhang, Fadi Li, Chong Li, Yongfu La, Futao Mo, Guoze Li, Yukun Zhang, Xiaolong Li, Qizhi Song, Yuan Zhao, Weimin Wang

**Affiliations:** ^1^College of Animal Science and Technology, Gansu Agricultural University, Lanzhou, China; ^2^Engineering Laboratory of Sheep Breeding and Reproduction Biotechnology in Gansu Province, Minqin Zhongtian Sheep Industry Co. Ltd., Minqin, China; ^3^The State Key Laboratory of Grassland Agro-ecosystems, College of Pastoral Agriculture Science and Technology, Lanzhou University, Lanzhou, China

**Keywords:** sheep, feed efficiency, residual feed intake, differentially expressed gene, single nucleotide polymorphism

## Abstract

In the genetic improvement of livestock and poultry, residual feed intake (RFI) is an important economic trait. However, in sheep, the genetic regulatory mechanisms of RFI are unclear. In the present study, we measured the feed efficiency (FE)-related phenotypes of 137 male Hu lambs, and selected six lambs with very high (n = 3) and very low (n = 3) RFI values and analyzed their liver transcriptomes. A total of 101 differentially expressed genes were identified, of which 40 were upregulated and 61 were downregulated in the low-RFI group compared with that in the high-RFI group. The downregulated genes were mainly concentrated in immune function pathways, while the upregulated genes were mainly involved in energy metabolism pathways. Two differentially expressed genes, *ADRA2A* (encoding adrenoceptor alpha 2A) and *RYR2* (ryanodine receptor 2), were selected as candidate genes for FE and subjected to single nucleotide polymorphism scanning and association analysis. Two synonymous mutations, *ADRA2A* g.1429 C > A and *RYR*2 g.1117 A > C, were detected, which were both significantly associated with the feed conversion rate. These findings provide a deeper understanding of the molecular mechanisms regulating FE, and reveal key genes and genetic variants that could be used to genetically improve FE in sheep.

## Introduction

In indoor sheep production systems, feed represents 65–70% of the cost of production (Zhang et al., 2017). Residual feed intake (RFI) and feed conversion ratio (FCR) are two conventional indexes to measure feed efficiency. The FCR is defined as the ratio of weight gain to feed intake over a specific time, whereas RFI is the difference between the predicted and actual feed intake, adjusted for the body size and performance of each animal (Zhang et al., 2017). Low-RFI animals produce fewer pollutants and generate less waste (Zhang et al., 2017), but have no effect on the body size, production, and weight of the animals. Low-RFI animals not only protect the environment, but also reduce feed costs, making RFI an economically relevant trait. Understanding the molecular mechanism of RFI will help to breed sustainable and profitable animals in agriculture (De Oliveira et al., 2018). The liver is a central controller of metabolism and energy balances, and is a major driver of whole animal oxygen consumption in cattle, chickens, and pigs. The liver has potential roles in the control of feed efficiency variation (Alhusseini et al., 2016; Xu et al., 2016; Ramayo-Caldas et al., 2018).

Factors that affect RFI include the digestion and metabolism of nutrients, body composition, body activity, energy output, and body temperature regulation (Zhang et al., 2017). Among them, energy metabolism is an important factor. Genes controlling energy metabolism affect the FCR of animals by regulating the energy metabolism of the body (Mckenna et al., 2019). In recent years, studies on RFI candidate genes have focused on pigs, cattle, and poultry (Gondret et al., 2017; Mukiibi et al., 2018; Zeng et al., 2018). Some progress has been made in revealing the molecular mechanisms underlying the RFI phenotype. Pig skeletal muscle transcriptome analysis of messenger RNAs (mRNAs) and microRNAs demonstrated downregulation of genes involved in mitochondrial energy metabolism and upregulation of those involved in skeletal muscle differentiation and proliferation in low-RFI animals compared with those in high-RFI, animals (Jing et al., 2015). In brown-egg dwarf hens with extreme RFI phenotypes, the duodenal transcriptome architecture identified significantly differentially expressed genes (DEGs) that are involved in metabolism, digestibility, energy homeostasis, and biosynthesis (Yi et al., 2015). In pigs, genome-level analyses of candidate genes and genetic markers for RFI using genome-wide associations and systematic genetic analyses revealed that single nucleotide polymorphisms (SNPs) in the genes encoding mitogen-activated protein kinase 5, peroxisomal biogenesis factor 7, and DS cell adhesion molecule could be markers associated with that for the RFI (Do et al., 2014). Gene functional annotation analysis suggested that the significant biological processes and pathways associated with RFI were gap junction formation, regulation of protein and lipid metabolism, the insulin signaling pathway, and inositol phosphate metabolism (Do et al., 2014). In Nellore cattle, a genome-wide association analysis of RFI and feed efficiency revealed markers located on chromosomes 4, 8, 14, and 21 in loci close to genes regulating ion transport and appetite (Santana et al., 2014). However, few studies have focused on the role of candidate genes in the process of RFI in sheep and as such, the genetic regulatory mechanisms of RFI are unclear.

In the present study, mRNA sequencing was used to determine the liver transcriptome and identify DEGs between sheep with extreme RFI values. We also explored the potential candidate genes that affect sheep RFI, which could lay the foundation for controlling in energy efficiency.

## Materials and Methods

### Ethics Statement

The experiments performed in the present study were executed according to the approved guidelines from the Regulation of the Standing Committee of Gansu People's Congress. The Ethics Committee of Gansu Agriculture University approved all the experimental protocols and the collection of samples.

### Animals and Tissues

In total, 598 male Hu lambs were purchased from Jinchang Zhongtian Sheep Industry Co. Ltd. (Jinchang, China) and transferred to Minqin Zhongtian Sheep Industry Co. Ltd. (Minqin, China). Healthy lambs at 56 days old, with good growth and available pedigrees, were selected randomly and immunized using a standardized program before weaning. The lambs were housed indoors in individual pens (0.8 × 1 m) until they were 180 days old. Briefly, the lambs were exposed to an acclimatization period of 14 days, during which the proportion of pellet feed in the diet was gradually increased by 7.1% per day, while the forage proportion was concurrently reduced, until they were only fed the pellet feed. The pre-test period was 10 days and the experimental period was 100 days, during which all the sheep were fed pellet feed, and had free access to food and water. Lambs were weighed in the morning before feeding, and at 0 and 100 days of the experimental recording period using calibrated electronic scales. Throughout the experiment the housing and feeding conditions, and the environment, were standardized. At the end of the experimental period, under the supervision of qualified veterinarians, blood samples (5 ml) were taken from the jugular vein of each sheep in the morning for subsequent DNA extraction. DNA was extracted using an EasyPure Blood Genomic DNA Kit (TransGen Biotech, Beijing, China). DNA was then dissolved in Elution Buffer (10 mM Tris hydrochloride, 1 mM ethylenediaminetetraacetic acid; pH 8.0) and stored at 20°C.

We collected the phenotype data of the experimental population at three different time, including 137 in the first batch, 207 in the second batch, and 254 in the third batch. We selected three high RFI sheep and three low RFI sheep from the first batch (137 male Hu lambs) to perform RNA sequencing. However, to expand the sample size of association analysis population, the phenotype of the other batches were measured after RNA sequencing. Within each RFI group, the sheep showed no difference in initial body weight (**Table 2**). A liver sample from each animal was obtained within 30 min after slaughter. The liver samples were frozen immediately using liquid nitrogen and then stored at -80°C before RNA isolation. All three batches were used for association analysis between genotypes and feed efficiency traits.

### Phenotypes

The phenotype of RFI was calculated using a linear regression model, using data on average daily gain (ADG), dry matter intake, and mid-test metabolic body weight (MBW) records for all the sheep.

The basic model (Zhang et al., 2017) used was:

$$\begin{array}{l} {\text{Y}}_{\text{j}}\text{= }\beta_{0}+\beta_{1}{\text{MBW}}_{\text{j}}+\beta_{\text{2}}{\text{ADG}}_{\text{j}}+{\text{e}}_{\text{j}}\\ \text{ADG = }\left(\text{FBW-IBW}\right)\text{/N}\\ \text{MBW = }{\left[\text{1/2}\times \left(\text{FBW+IBW}\right)\right]}^{0.75} \end{array}$$

where Y_j_ is the dry matter intake of the j^th^ animal, β_0_ is the regression intercept, β_1_ is the regression coefficient on MBW, β_2_ is the regression coefficient on ADG, and e_j_ is the uncontrolled error of the j^th^ animal, FBW is final body weight, IBW is the initial body weight, and N is the test duration (days).

### RNA Preparation and Sequencing

The Trizol reagent (Invitrogen, Waltham, MA, USA) was used to extract total RNA, according to the manufacturer's protocols. For each RNA-sequencing (RNA-seq) sample, a RNA sequencing library was constructed using a TruSeq^®^ Stranded Total RNA Sample Preparation kit (Illumina^®^, San Diego, CA, USA), performed according to the kit's manual. After quality control of the libraries, a HiSeq2500 instrument (Illumina) was used to sequence all the libraries.

### Analyses of RNA-Seq Data

First, fqall2std.pl was used to transform the RNA-Seq data from the Illumina fastq format to the standard Sanger fastq format. Next, the Tophat-Cufflinks pipeline (Beijing Novogene Science and Technology Co., Ltd) was used to process the data. The reference genome of *Ovis aries* (Oarv4.0) and gene model annotation files were obtained from Ensembl. The reads were aligned to the genome using the build index with bowtie version 2.0.6. and TOPHAT (version 2.0.9), using the option: library-type fr-firststrand. Transcriptome assembly was performed using Cufflinks (version 2.1.1), and gene expression analysis was executed using the Cuffdiff script (in Cufflinks) with the option: classic-fpkm. The fragments per kilobase of exon per million fragments mapped value was calculated to represent the expression level of each gene, based on the fragment length and read counts that mapped to a fragment. Finally, q ≤ 0.05 was set as the threshold for DEG selection. Quantitative real-time reverse transcription PCR (qRT-PCR) was used to validate the DEGs, we selected six sheep with the highest RFI (named the high-RFI group) and the six sheep with lowest RFI (low-RFI group) from the first batch (137 male Hu lambs) for extracting total RNA. Total complementary DNA (cDNA) was synthesized using a reverse transcriptase kit (Takara, Dalian, China). QPCR was performed using a SYBR green assay (Takara Biotechnology) on a Roche LightCycler 480 (Roche Applied Science, Mannheim, Germany). The specific quantitative primers for five transcripts are listed in **Supplementary Material Table S1**. Each 20-µl reaction contained 6.4 µl of H_2_O, 0.8 µl of each primer, 2 µl of cDNA, and 10 µl of 2 × SYBR Green PCR Master Mixture (Takara Biotechnology). The conditions were as follows: An initial single cycle (95°C for 3 min), followed by 40 cycles (95°C for 15 s; the optimized annealing temperature for 15 s; and 72°C for 20 s) and a final extension step at 72°C for 5 min. All amplifications were followed by dissociation curve analysis of the amplified products. Gene expression levels were normalized to that of the *ACTB* gene (encoding beta actin) to determine the relative expression using the 2^ΔΔCt^ value method. The average change in the cycle threshold (ΔCt) value of the low-RFI group was used as the reference to calculate the ΔΔCt value for each gene (Livak and Schmittgen, 2001), and the expression difference between the two groups was calculated using Student's t-test.

### Kyoto Encyclopedia of Genes and Genomes Enrichment Analysis

The Kyoto Encyclopedia of Genes and Genomes (KEGG) database contains resources that allow us understand gene functions in a biological system, for example at the cell, organism, and ecosystem levels. KEGG uses molecular level information, especially that generated by high-throughput experimental technologies, such as the large-scale molecular datasets generated by genome sequencing (http://www.genome.jp/kegg/). The statistical enrichment of DEGs in KEGG pathways was tested using the KOBAS software.

### Single Nucleotide Polymorphism Identification and Genotyping

We selected two genes (*ADAR2A* and *RYR2*) related to adrenaline pathway from 101 DEGs as candidate genes for SNP scanning. The SNPs in these two genes were identified by sequencing the PCR products that were amplified using mixed DNA samples from the Hu Sheep. The genomic DNA sequences of the candidate genes were used to design specific PCR primers (**Supplementary Material Table S2**). The PCR reaction for sequencing was performed in a volume of 25 µl, containing 10 × PCR buffer, 0.35 µM primers, 87.5 µM dNTPs, 50 ng of genomic DNA, and 1.25 of UTaq DNA Polymerase (TransGen Biotech) using the following thermocycling conditions: 5 min at 94°C, followed by 30 s at 94°C, 30 s at 50–60°C, and 30 s at 72°C (35 cycles), with a final extension incubation for 5 min at 72°C. Finally, competitive allele specific FRET-based PCR (KASPar) assays (LGC Genomics, Hoddesdon, UK) were used to genotype the SNPs identified within the candidate genes according to a previously published method (Smith and Maughan, 2015). The primers used by KASPar are shown in **Supplementary Material Table S3**. KASPar genotyping was developed using competitive allele-specific PCR and allows bi-allelic SNP scoring. To the DNA samples, the universal KASP Master Mix and SNP-specific KASP Assay mix (LGC genomics) were added. At the end of the thermal cycling, an end-point fluorescent reading was carried out. For allelic discrimination, competitive annealing of two allele-specific forward primers was performed. Each primer had a unique tail sequence that corresponded to a distinct labeled FRET cassette in the Master Mix. One was labeled with a HEX^™^ dye and the other with a FAM^™^ dye (Faucher et al., 2016).

### Statistical Analysis

The general linear model program was used to perform the association analysis between genotypes and FCR. The specific model was defined as follows:

$$\begin{array}{l} {\text{Y}}_{\text{ij}}=\mu +{\text{ G}}_{\text{i}}{\text{+ B }}_{\text{j}}+\varepsilon_{\text{ij}}\\ \text{FCR = average daily feed intake/ADG}. \end{array}$$

In this model, Y_ij_ represents the phenotypic observation of FCR, µ is the mean, G_i_ is the effect of the i_th_ genotypes, B_j_ is the batch effect and ε_ij_ is the residual corresponding to the trait's observed value, *P* < 0.05 was considered to indicate statistical significance. The boxplots of FCR for different genotypes were computed using the ggplot packages in the R software.

## Results

### Animal Performance Traits

Male Hu lambs (n = 137) were grown from 56 to 180 days old. The individual body weight (BW) and FI were measured and the ADG, FCR, and RFI were calculated (**Table 1**). From this data, sub-groups were selected from this data that had low or high RFI values i.e., the low-RFI and high-RFI groups. Notably, there was no significant difference in ADG between the two groups (**Table 2**). The high-RFI group had RFI and FCR values of 0.20 ± 0.02 (kg/day) and 5.62 ± 0.38, respectively, compared with -0.25 ± 0.05 (Kg/day) and 3.92 ± 0.25 in the more efficient low-RFI group (*P* < 0.05 for both FCR and RFI; **Table 2**). In the low-RFI group, a reduced FI resulted in a lower FCR (*P* = 0.019). We also noted there was no significant difference in average MBW between the two groups. Similar results were obtained in the other batches.

**Table 1:** Descriptive statistics of all the experimental animals.

Trait	N	Min	Max	Mean	SD
FCR (kg of FI/kg of BW gain)	137	3.63	7.09	4.90	0.59
RFI (kg/day)	137	-0.31	0.22	0.00	0.09
FI (kg/day)	137	0.72	1.72	1.22	0.18
ADG (kg/day)	137	0.17	0.33	0.25	0.03
Initial BW (kg)	125	1.42	18.99	2.75	1.55
Final BW (kg)	137	15.15	36.70	24.72	3.95
Metabolic BW (kg0.75)	137	9.82	16.89	13.11	1.33
BW before slaughter (kg)	137	29.15	53.20	40.37	4.48

FCR, feed conversion ratio; RFI, residual feed intake; FI, average daily feed intake over the assessed feeding period; ADG, average daily gain over the assessed feeding period; BW, body weight.

**Table 2:** Performance of the male Hu lambs used in RNA sequencing.

	High-RFI	Low-RFI	p-value
n	3	3	
FCR (kg of FI/kg of BW gain)	5.62 ± 0.38	3.92 ± 0.25	0.004
RFI (kg/day)	0.20 ± 0.02	-0.25 ± 0.05	0.001
FI (kg/day)	1.48 ± 0.14	1.05 ± 0.13	0.019
ADG (kg/day)	0.26 ± 0.02	0.27 ± 0.02	0.827
Initial BW (kg)	25.43 ± 3.75	25.76 ± 2.09	0.900
Final BW (kg)	38.60 ± 4.35	39.10 ± 2.69	0.875
Metabolic BW (kg^0.75^)	13.44 ± 1.28	13.59 ± 0.74	0.878
BW before slaughter (kg)	41.18 ± 4.06	42.36 ± 1.98	0.678

RFI, residual feed intake; FCR, feed conversion ratio; FI, average daily feed intake over the assessed feeding period; ADG, average daily gain over the assessed feeding period; BW, body weight. P-value as calculated by a t-test.

### RNA Sequencing Data Mapping and Annotation

**Figure 1:**
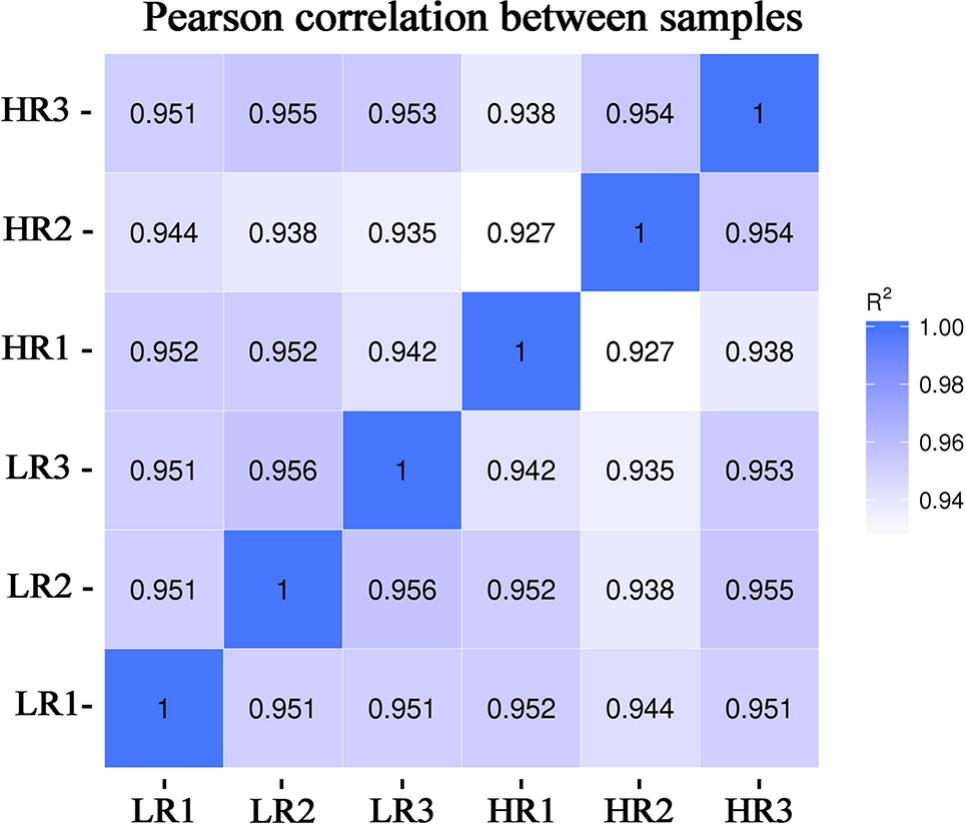
Chart of the expression correlation between samples. Data for six samples, comprising three samples with very high-RFI values and three samples with very low-RFI values, are presented. The horizontal coordinate of the correlation coefficient between samples is log10(FPKM+1) of sample 1, the ordinate is log10(FPKM+1) of sample 2, and R2 is the square of Pearson's correlation coefficient. FPKM—fragments per kilobase of exon per million fragments mapped.

From the livers of the low-RFI and high-RFI groups, six cDNA libraries were sequenced and Pearson's correlation coefficient among the samples ranged from 0.92 to 0.96, indicating high similarity of the expression patterns between the samples (**Figure 1**). After sequencing, a total of 370,689,678 raw reads were obtained from the high-RFI group and 334,871,310 from the low-RFI group. To test the quality of the RNA-seq data, we performed a series of quality control analyses. First, the Q30 i.e., the percentage of the total number of bases with a Phred score greater than 30, of the reads in all samples ranged from 91.5 to 93.22%. The average GC content of the six libraries was 50.24%. After filtering out the adaptor sequences, empty sequences, and low-quality sequences, 352,944,368 (high-RFI), and 328,070,686 (low-RFI) clean reads were obtained (**Supplementary Material Table S4**). The clean reads were then used to perform a biological information analysis. The mapping rate to the *O. aries* reference genome (Oarv4.0) of the clean data was between 81.07 and 83.03% for all six samples (**Supplementary Material Table S5**).

### Differentially Expressed Genes Between Low- and High-RFI Groups

RNA-seq detected a total 22,801 genes in the livers of all six individuals, among which 744 genes were differentially expressed. DEGs were identified using the criteria of at least a 2-fold difference in expression and a p-value less than 0.05 (|log2FC|≥ 1, *P* < 0.05). Among the 744 DEGs, 101 had a q-value ≤ 0.05. Of the 101 DEGs, 40 were upregulated and 61 were downregulated in the low-RFI group (**Supplementary Material Table S6**). **Table 3** shows the top 20 DEGs, representing the top 10 upregulated genes and the top 10 downregulated genes in the low-RFI group compared with that in the high-RFI group.

**Table 3:** Top 20 liver DEGs between male Hu lambs with low- and high-RFI values.

Gene	FPKM		Log_2_(FC)	q-value	Full name
	Low-RFI	High-RFI			
SPP1	3.43	0.33	3.38	1.53E-02	Secreted phosphoprotein 1
WNT2B	4.25	0.70	2.59	8.28E-03	Wnt family member 2B
FCGBP	1.19	0.25	2.22	8.28E-03	Fc fragment of IgG binding protein
ACTG2	3.39	0.89	1.92	8.28E-03	Actin, gamma 2, smooth muscle
SLITRK4	1.41	0.38	1.88	4.38E-02	SLIT and NTRK-like family, member 4
RUNDC3B	8.73	2.41	1.86	8.28E-03	RUN domain containing 3B
NEXN	1.96	0.55	1.82	8.28E-03	Nexilin
SHISA3	0.87	0.25	1.81	2.67E-02	Shisa family member 3
ME1	8.48	2.43	1.81	8.28E-03	Malic enzyme 1
PPARGC1A	20.99	6.41	1.71	8.28E-03	PPARG coactivator 1 alpha
ADRA2A	0.65	5.21	-2.99	8.28E-03	Adrenoceptor alpha 2A
NRCAM	0.59	3.06	-2.38	8.28E-03	Neuronal cell adhesion molecule
WWC1	11.02	57.49	-2.38	8.28E-03	WW and C2 domain containing 1
TNC	28.68	149.08	-2.38	8.28E-03	Tenascin C
RFC3	0.36	1.80	-2.30	8.28E-03	Replication factor C subunit 3
DIRAS3	3.84	18.61	-2.28	8.28E-03	DIRAS family gtpase 3
IL1R2	2.39	11.14	-2.22	8.28E-03	Interleukin 1 receptor
TCAF2	3.40	15.43	-2.18	8.28E-03	TRPM8 channel-associated factor 2
PTGER3	1.29	5.47	-2.08	2.16E-02	Prostaglandin E receptor 3
RIMS1	0.61	2.34	-1.94	2.16E-02	Regulating synaptic membrane exocytosis 1

DEG, differentially expressed gene; RFI, residual feed intake; Log_2_FC, log_2_(low-RFI/high-RFI); FPKM, fragments per kilobase of exon per million fragments mapped.

**Figure 2:**
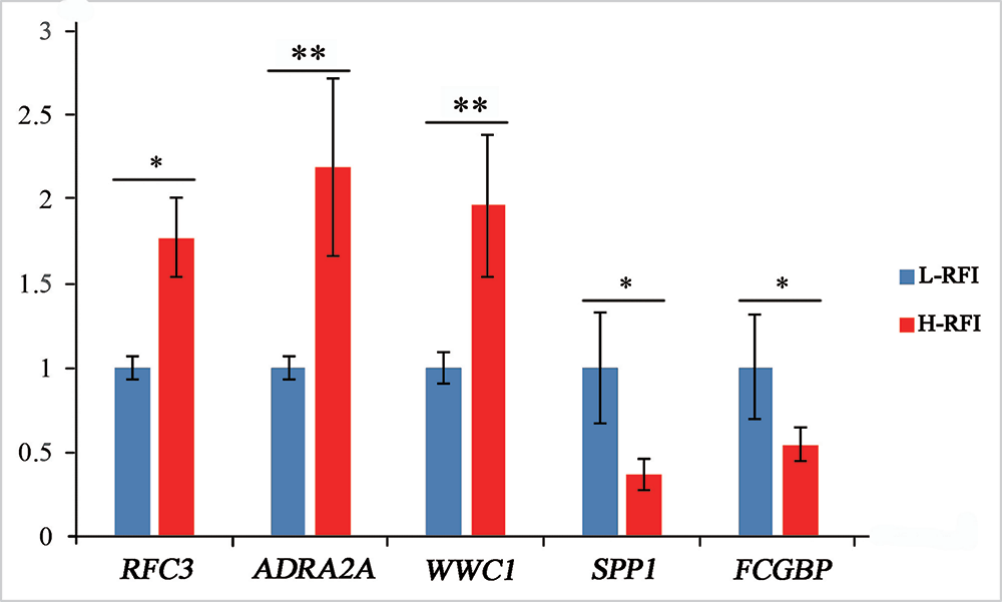
Validation of differentially expressed genes (DEGs) using quantitative real-time reverse transcription PCR (qRT-PCR). The qRT-PCR measurements of the expression of *RFC3*, *ADRA2A*, *WWC1*, *SPP1*, and *FCGBP* mRNA transcripts were analyzed using the ΔΔCt method; *significant difference between the high-RFI and low-RFI groups (*P* < 0.05), **very significant difference between the high-RFI and low-RFI groups (*P* < 0.01).

Five DEGs were selected for qRT-PCR analysis to validate their differential expression. The expression levels of *WWC1* (encoding WW and C2 domain containing 1), *RFC3* (encoding replication factor C subunit 3), and *ADRA2A* (encoding adrenoceptor alpha 2A) mRNA were lower in the low-RFI liver compared with that in the high-RFI liver, whereas the expression of *SPP1* (encoding secreted phosphoprotein 1) and *FCGBP* (encoding Fc fragment of IgG binding protein) mRNA was higher in the low-RFI liver (**Figure 2**). The expression levels of five of the selected genes (*WWC1*, *RFC3*, *FCGBP*, *SPP1*, and *ADRA2A*) were significantly different between the high- and low-RFI groups, showing the qRT-PCR analyses confirmed the RNA-seq data.

### Pathway Analysis of Differentially Expressed Genes

**Figure 3:**
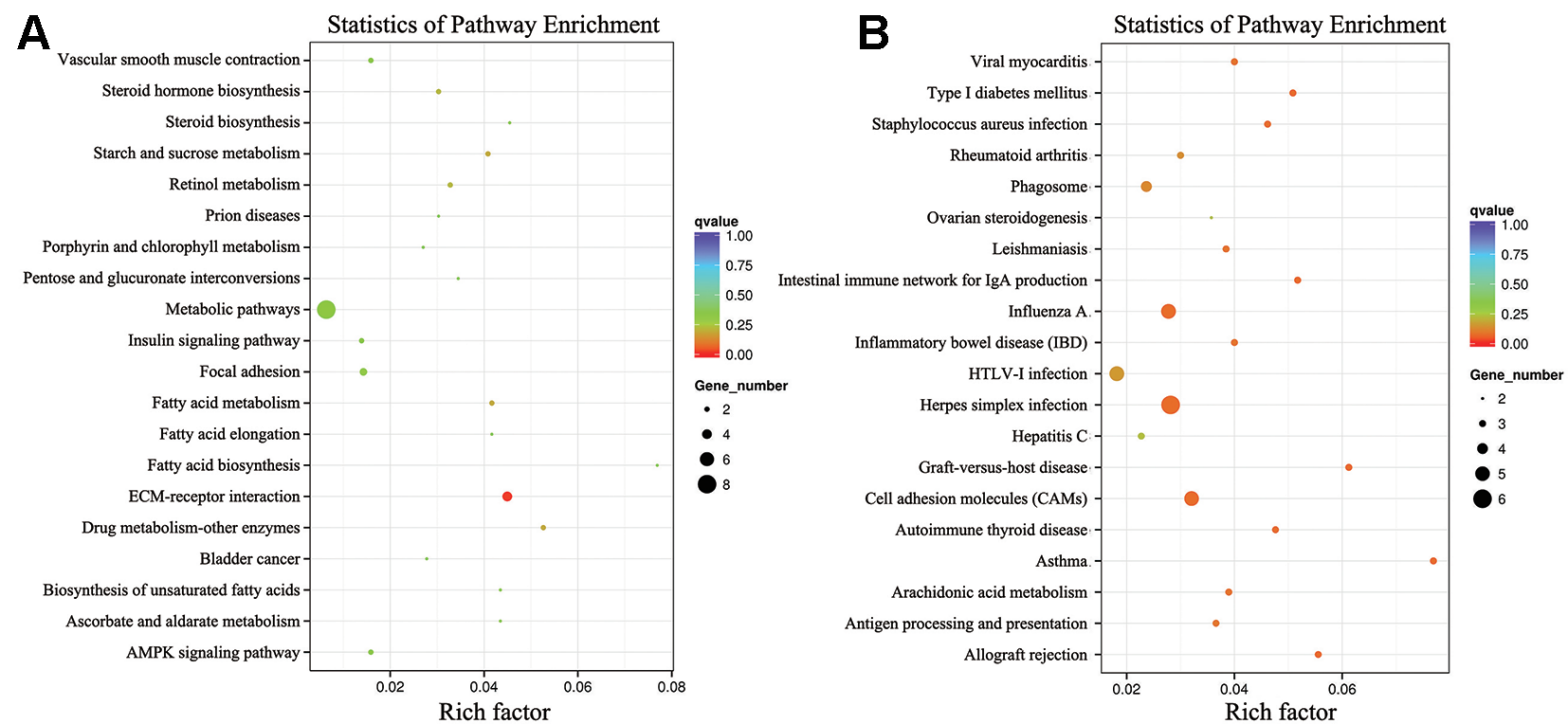
Kyoto Encyclopedia of Genes and Genomes (KEGG) classification of differentially expressed genes (DEGs) between the high- and low-RFI groups. **(A)**: Upregulated genes; **(B)**: Downregulated genes.

To further elucidate the functions of the DEGs, we performed KEGG pathway enrichment analyses. The KEGG pathway analysis of the DEGs between the low-RFI and high-RFI groups revealed that the downregulated genes were mainly involved in immune function pathways, including *DQA* (major histocompatibility complex, class II, DQ alpha 1), *RFC3*, *RYR2* (ryanodine receptor 2), and *PTGER3* (prostaglandin E receptor 3). The upregulated genes were mainly involved in metabolic pathways, including *SPP1*, *FCGBP*, *PPARGC1A* (PPARG coactivator 1 alpha), and *ME1* (malic enzyme 1) (**Figure 3**).

### Single Nucleotide Polymorphism Scanning of the *ADRA2A* and *RYR2* Genes

**Figure 4:**
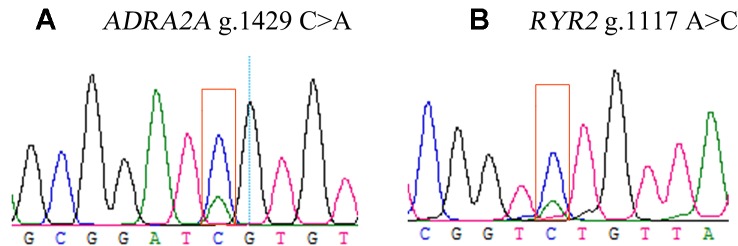
Sequencing peak images for ovine *ADRA2A* **(A)** and *RYR2* **(B)** genes.

**Figure 5:**
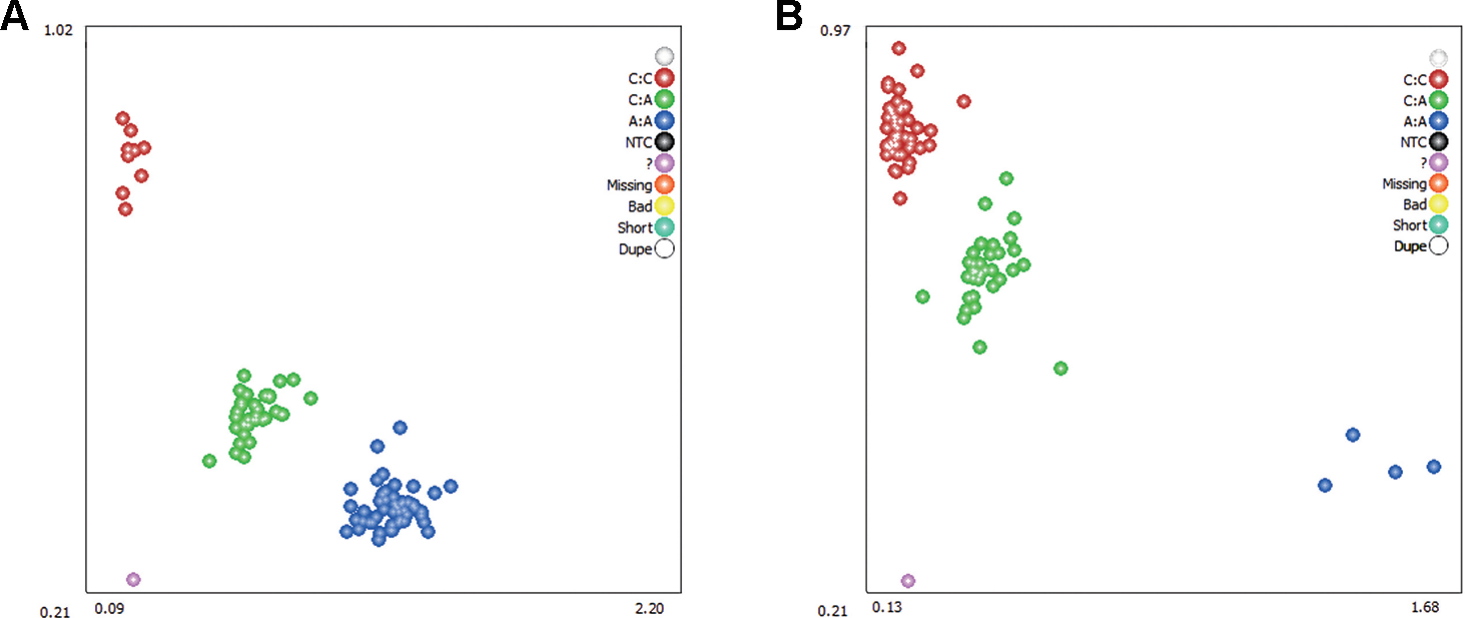
Genotyping of ovine *ADRA2A* g.1429 C > A **(A)** and *RYR2* g.1117 A > C **(B)** mutations using Kaspar technology. Note: The red, green, and blue dots represent the three genotypes, respectively; while the purple dots indicate genotyping failure.

Two DEGs, *ADRA2A* and *RYR2*, were selected as candidate genes related to feed efficiency. Two synonymous mutations, g.1429 C > A and g.1117 A > C were discovered in ovine the *ADRA2A* and *RYR2* genes, respectively, by sequencing the PCR products, which were amplified using mixed Hu Sheep DNA samples (**Figure 4**). The genomic DNA of these two candidate genes was used to design specific PCR primers (**Supplementary Material Table S2**). These two SNPs were genotyped using KASPar assays, which generated three genotypes for both genes: AA, CC, and AC (**Figure 5**).

### Association Analysis of *ADRA2A* and *RYR2* Genes With the Feed Efficiency

The association of SNPs in the *ADRA2A* and *RYR2* genes with the FCR in the enlarged (n = 561) experimental population was studied. The result of the association analysis showed that the novel polymorphisms *ADRA2A* g.1429 C > A and *RYR2* g.1117 A > C were both significantly associated with FCR (*P* < 0.05 or *P* < 0.01). Lambs carrying the CC genotype of the *ADRA2A* g.1429 C > A mutation had significantly lower FCR than those carrying the AA and CA genotypes (0.51 and 0.47, respectively; *P* < 0.01) (**Table 4**). Lambs carrying the AA genotype of the *RYR2* g.1117 A > C mutation had a significantly larger FCR than those carrying the CC and CA genotypes (0.33 and 0.38, respectively; *P* < 0.05) (**Table 4**).

**Table 4:** Association analysis of ovine *ADRA2A* g.1429 C > A and *RYR2* g.1117 A > C single nucleotide polymorphisms in the experimental population.

Gene/loci	Genotype	No.	FCR
*ADRA2A* g.1429 C > A	CC	37	4.67 ± 0.69B
	CA	215	5.18 ± 0.92A
	AA	309	5.14 ± 0.93A
*RYR*2 g.1117 A > C	CC	327	5.13 ± 0.56b
	CA	207	5.08 ± 0.65b
	AA	27	5.46 ± 0.66a

Feed conversion rate (FCR) is indicated by the mean + SD. In the same column, there were significant differences between numerical data labeled with different lowercase letters (P < 0.05), and between data labeled with different capital letters (P < 0.01).

## Discussion

Two groups of male Hu lambs were phenotyped that had either high- or low-RFIs. The RFI, FI, and FCR values in the high-RFI lambs were significantly higher than those in the low-RFI lambs; however, there were no differences in the ADG, initial BW, final BW, MBW, and BW before slaughter, which was in agreement with the findings of Zhang et al. (2017). This result indicated that increased feed efficiency in sheep could result from the selection of RFIs by reducing feed consumption without affecting their growth performance. Therefore, RFI could be used as an ideal breeding index for feed efficiency traits in sheep.

The liver has a pivotal role in host physiology, carrying out most amino acid, carbohydrate, and lipid metabolism; detoxification; ketogenesis; urea synthesis; and albumin and glutathione synthesis; and is important in innate immunity (Rojas et al., 2015). In the present study, 101 DEGs were identified (q ≤ 0.05), including 61 downregulated genes between the low- and high-RFI animals, which are mainly concentrated in metabolic pathways. The results showed that metabolism-related genes were highly expressed in the liver tissues of the lambs with low-RFI, and the metabolic efficiency of the liver tissues of the lambs with low-RFI was improved, thereby improving feed efficiency.

The immune response process is another important factor affecting the efficiency of animal feed (Kyriazakis et al., 1998; Buyse and Decuypere, 2015). The immune system uses a lot of energy, for example, in mice that were immunized with a relatively benign antigen, metabolic heat production and oxygen consumption increased by 20–30% (Johnson et al., 2013). When an animal is diseased, the body will produce an immune response and the amount of food intake will be reduced. Immune defense and immune response are important mechanisms for animals to maintain their health. Energy metabolism is closely connected with productivity traits. The underlying mechanisms that control the immune defense or immune response may also affect the mechanisms that regulate energy metabolism (Verhagen, 1987). Liu et al. (2016) studied the whole blood expression profile of high and low-RFI pigs and identified genes involved in the immune system. In addition, Integrated Pathway Analysis Database-based disease-associated gene enrichment analysis suggested that genes involved in several diseases were overrepresented among the DEGs (Liu et al., 2016)(Liu et al., 2016). In the present study, 40 DEGs were upregulated between the low- and high-RFI animals, which were mainly associated with immune pathways, including asthma, cell adhesion molecules, livestock influenza, the production of IgA intestinal immune network, type 1 diabetes, and inflammatory bowel disease, which showed that the high-RFI lambs had a heightened immune response. By stimulating the immune response, the body's energy consumption increases, thus lowering feed efficiency. Therefore, in the process of feeding, ensuring body health is an important basis to maximize the growth performance of animals.

Two DEGs, *ADRA2A*, and *RYR2* participate in the adrenaline pathway and may regulate energy metabolism through the secretion of adrenaline, thus affecting feed efficiency. Therefore, the two genes were selected as candidate genes related to feed efficiency. Two synonymous mutations, g.1429 C > A and g.1117 A > C were discovered in the ovine *ADRA2A* and *RYR2* genes, respectively. Association analysis revealed that these two polymorphisms had a significant effect on feed efficiency. ADRA2A, a member of the G protein-coupled receptor superfamily, regulates neurotransmitter release from adrenergic neurons and sympathetic nerves in the central nervous system (Manca et al., 1997; Fagerholm et al., 2004). ADRA2A is a regulator of catecholamines, which have been reported as associated with energy metabolism, and gene-regulated catecholamine release may play an important role in obesity (Lima et al., 2007). ADRA2A is also related to fat metabolism (Rosmond et al., 2010), and is implicated in several functions in the central nervous system, cardiovascular system, neurotransmitter release, platelet aggregation, blood pressure, insulin secretion, and lipolysis (Kaabi et al., 2016). Additionally, catecholamine-stimulated whole body lipolysis and lipolysis in subcutaneous adipocytes are blunted in obesity (Blaak et al., 1994; Large and Arner, 1998), thereby limiting lipid mobilization and favoring fat accumulation, which suggests that ADRA2A might be involved in fat and energy metabolism (Lima et al., 2007). Energy metabolism and fat metabolism are the key factors affecting the feed conversion rate of livestock and poultry (Kaewpila et al., 2018). However, the relationship between the *ADRA2A* gene and feed efficiency in animals has not been reported previously. In our study, the *ADRA2A* gene was downregulated in the low-RFI group. Moreover, polymorphism g.1429 C > A in this gene was significantly associated with FCR. Therefore, we speculated that *ADRA2A* might affect FCR by changing energy metabolism and fat metabolism.

RYR2 is the main sarcoplasmic reticulum Ca^2+^ release channel in ventricular myocytes (Jiang et al., 2004). RYR2 regulation causes contractile dysfunction in a variety of cardiac pathologies (Bround et al., 2016). In particular, abnormal RYR2 activity contributes to sarcoplasmic reticulum Ca^2+^ mishandling, arrhythmias, and contractile dysfunction in failing hearts (Zima et al., 2014). In the present study, we found that *RYR2* was downregulated in the low-RFI group, and that the gene has a 1117 A > C mutation. Carrying the AA genotype of the *RYR2* mutation resulted in a significantly larger FCR than that in those carrying the CC and CA genotypes. Therefore, RYR2, by regulating the excitement and contraction of skeletal muscle and cardiac muscle cells, affects the body's metabolic rate, which participates in the regulation of the sheep FCR; however, its regulation mechanism requires further study.

## Conclusions

We identified 101 DEGs (40 upregulated and 61 downregulated) in liver of Hu lambs with different RFIs. Certain metabolism-related genes were upregulated between the high- and low-RFI groups, while downregulated genes were enriched in immune pathways. Importantly, two polymorphisms detected in DEGs *ADAR2A* and *RYR2* were significantly associated with feed efficiency. These results provide new insights into the molecular mechanism of feed efficiency and identified valuable candidate genes for marker-assisted selection to improve feed efficiency in sheep production.

## Data Availability Statement

The RNA-Seq Bioproject data are accessible at National Center for Biotechnology Information (NCBI) Bioproject under accession number PRJNA547871. All RNA sequencing data have been submitted to the NCBI Sequence Read Archive under accession numbers SRR9291141, SRR9301148, SRR9301110, SRR9302163, SRR9302192, and SRR9302375.

## Ethics Statement

The animal study was reviewed and approved by Standing Committee of Gansu People's Congress and The Ethics Committee of Gansu Agriculture University.

## Author Contributions

DZ and WW conceived the study. DZ, XZ, WW, FM, and YL contributed to growth performance and feed intake. DZ, XZ, FL, CL, GL, YKZ, XL, QS, YZ, and WW contributed to sample collection and prepared biological samples. DZ, XZ, and WW analyzed the data. DZ wrote the paper. DZ and WW revised the paper. All authors read and approved the final manuscript.

## Funding

This work was supported by the National Natural Science Foundation of China (grant no. 31760651 and 31560625) and the Earmarked Fund for China Agriculture Research System (CARS-38).

## Conflict of Interest

Authors XZ and FL were employed by company Minqin Zhongtian Sheep Industry Co. Ltd., China. The remaining authors declare that the research was conducted in the absence of any commercial or financial relationships that could be construed as a potential conflict of interest.
